# Transgenesis in *Strongyloides* and related parasitic nematodes: historical perspectives, current functional genomic applications and progress towards gene disruption and editing

**DOI:** 10.1017/S0031182016000391

**Published:** 2016-03-22

**Authors:** J. B. LOK, H. SHAO, H. C. MASSEY, X. LI

**Affiliations:** Department of Pathobiology, School of Veterinary Medicine, University of Pennsylvania, 3800 Spruce Street, Philadelphia, PA 19104, USA

**Keywords:** *Strongyloides*, nematode, *Caenorhabditis*, transgenesis, microinjection, chromosomal integration, transposon, CRISPR/Cas9

## Abstract

Transgenesis for *Strongyloides* and *Parastrongyloides* was accomplished in 2006 and is based on techniques derived for *Caenorhabditis elegans* over two decades earlier. Adaptation of these techniques has been possible because *Strongyloides* and related parasite genera carry out at least one generation of free-living development, with adult males and females residing in soil contaminated by feces from an infected host. Transgenesis in this group of parasites is accomplished by microinjecting DNA constructs into the syncytia of the distal gonads of free-living females. In *Strongyloides stercoralis*, plasmid-encoded transgenes are expressed in promoter-regulated fashion in the F1 generation following gene transfer but are silenced subsequently. Stable inheritance and expression of transgenes in *S. stercoralis* requires their integration into the genome, and stable lines have been derived from integrants created using the *piggyBac* transposon system. More direct investigations of gene function involving expression of mutant transgene constructs designed to alter intracellular trafficking and developmental regulation have shed light on the function of the insulin-regulated transcription factor Ss-DAF-16. Transgenesis in *Strongyloides* and *Parastrongyloides* opens the possibility of powerful new methods for genome editing and transcriptional manipulation in this group of parasites. Proof of principle for one of these, CRISPR/Cas9, is presented in this review.

## INTRODUCTION

Transgenesis in *Strongyloides* spp. and in the related genus *Parastrongyloides* has been achieved largely by appropriation of culture methods (Stiernagle, 2006) and techniques for gene transfer by gonadal microinjection in *Caenorhabditis elegans* (Evans, 2006). *Strongyloides* and related genera are uniquely suited to adaptation of these methods because their life cycles include one or more generations of free-living male and female worms and their progeny which are similar in size, overall body plan and culture biology to *C. elegans* hermaphrodites and larvae. The architectures of gonads in free-living female *Strongyloides* and *Parastrongyloides* while differing in some fundamental respects (Kulkarni *et al.* 2015), are similar enough to that of *C. elegans* that infusion of plasmid-based vectors into their syncytia transforms proportions of germ cells that are sufficient to enable robust experiments on developing transgenic larvae and parasitic adults (Lok, 2012). In *C. elegans*, multi-copy transgene arrays generated by gonadal microinjection of plasmid-based vectors are inherited and expressed stably through many generations of passage. Although assembly of plasmid vectors into arrays has not been formally proven for *Parastrongyloides*, this parasite also inherits and expresses plasmid-based transgenes through multiple generations following transformation (Grant *et al.* 2006*a*). By contrast, *Strongyloides* spp. do not express transgenes encoded in plasmid-based vectors beyond the F1 generation following transformation (Lok, 2012). However, transposon-mediated chromosomal integration of transgene constructs in *Strongyloides ratti* enables the establishment of transgenic lines of parasite that uniformly transmit and express these constructs over an indefinite number of host and culture passages (Lok, 2013).

This review will recount the development of both transient and stable transgenesis in *C. elegans* and discuss the specifics of adapting these methods to *Strongyloides* spp. and *Parastrongyloides*. It will go on to discuss current applications of transgenesis into investigations of gene function in *Strongyloides stercoralis*. These encompass the use of transcriptional reporters to ascertain anatomical and temporal patterns of gene expression and the deployment of a dominant interfering gene construct to infer developmental and morphogenetic function of *Ss-daf-16*, the orthologue of the eponymous dauer regulatory transcription factor in *C. elegans*. The review will end with an assessment of future research priorities in the area of transgenesis in *Strongyloides* and other parasitic nematodes. Not least among these is to capitalize on the opportunity afforded by robust techniques for transgenesis to deploy contemporary methods for gene disruption and editing and for experimental manipulation of transcription levels in genes of interest in *Strongyloides* and *Parastrongyloides*. This discussion will focus on clustered regularly interspaced short palindromic repeats (CRISPR)/Cas9-associated methods, which are rapidly gaining traction in *C. elegans* science (Frokjaer-Jensen, 2013; Lo *et al.* 2013; Waaijers *et al.* 2013) and will include new data from the authors' laboratory that provide proof-of-principle for targeted gene mutation by CRISPR/Cas9 in *S. stercoralis*. The review will end by articulating the imperative to extend transgenesis to other groups of medically important parasitic nematodes, most likely by developing alternative methods of gene transfer that do not require access to the adult female and do not rely upon microinjection.

## THE DEVELOPMENT OF TRANSGENESIS IN *STRONGYLOIDES*

### Background on transgenesis in C. elegans

Current methods for transgenesis in *Strongyloides* and related parasitic nematodes are based on methods developed for *C. elegans* beginning in 1982 with the first successful transformation of germ cells with tRNA (Kimble *et al.* 1982). Heritable DNA transformation of *C. elegans* was achieved shortly thereafter by microinjection of plasmid-based constructs into the gonadal syncytium of adult hermaphrodites (Stinchcomb *et al.* 1985), a process mainly resulting in assembly of transgene sequences into extrachromasomally inherited multi-copy arrays (Mello and Fire, 1995). The advent of selectable transgene-encoded markers (Fire, 1986) allowed lines of *C. elegans* transformed with these arrays to be perpetuated, and further technical improvements in creating lines with extrachromasomal arrays and in integrating these arrays into the chromosome (Mello *et al.* 1991) solidified transgenesis by gonadal microinjection as a method to investigate the biology of *C. elegans*. Subsequently, the potentially confounding effects of transgene overexpression from high-copy arrays prompted the search for methods for low- or single copy transgene integration into the *C. elegans* chromosome. In this vein, biolistic transfer of DNA constructs (Praitis *et al.* 2001) was found to give a higher frequency of spontaneous low-copy chromosomal integration of transgenes than the exceedingly low frequency of these integrations that result from gonadal microinjection of DNA (Mello and Fire, 1995). More recently, a system for single-copy insertion of transgenes mediated by the Mos1 transposon was devised for *C. elegans* (Frokjaer-Jensen *et al.* 2008, 2012). Reviews of essential aspects of *C. elegans* transformation by gonadal microinjection and by biolistics, along with detailed laboratory protocols for these methods, are contained in WormBook (Evans, 2006; Schweinsberg and Grant, 2013).

### The free-living female as a target for heritable transgenesis in Strongyloides and Parastrongyloides

Ready access to free-living females of *Strongyloides* and *Parastrongyloides* has been the key to adapting the technique of gonadal microinjection as a means of DNA transfer to these organisms. *Strongyloides* and related genera are unique among the parasitic nematodes in their capacity to undertake one or more generations of free-living development, with free-living males and females and their larval progenitors and progeny living in soil contaminated with host feces (Viney and Lok, 2015). In most species of *Strongyloides*, including *S. stercoralis* and *S. ratti*, the species in which transgenesis has been achieved, only one generation of free-living development occurs and all progeny of free-living males and females develop to infective third-stage larvae (iL3) that must infect a host in order to continue development. By contrast, *S. planiceps*, a parasite of cats, can execute up to nine sequential free-living generations with each exhibiting a progressive decline in fecundity (Yamada *et al.* 1991; Viney and Lok, 2015), and *Parastrongyloides trichosuri*, a parasite of the Australian brush-tailed possum, can develop indefinitely as a free-living nematode, with the number of free-living generations limited only by increasing population density and declining food levels in the worm's immediate environment. Similar to dauer switching in *C. elegans*, these limiting factors favour development by *P. trichosuri* to iL3 and subsequent host invasion over further free-living development (Grant *et al.* 2006*b*).

In general, the free-living females of *Strongyloides* and *Parastrongyloides* are strikingly similar to hermaphrodites of *C. elegans* in their overall body plans and in their adaptability to laboratory culture. Like *C. elegans* hermaphrodites, they measure approximately one millimetre in length; their body walls are transparent, and their ovaries are didelphic, with two recurved arms converging on the vulva at midbody (Viney and Lok, 2015). Free-living adults and their progeny may be maintained on standard plates of Nematode Growth Medium agar with lawns of *Escherichia coli* OP50 or HB101 bacteria. Survival and fecundity of these free-living stages may be enhanced by adding judicious quantities of host fecal solids or extracts thereof to their culture plates (Ashton *et al.* 1998; Nolan *et al.* 2004; Grant *et al.* 2006*b*), and *Strongyloides* spp., which are derived from culture of host feces, invariably carry fecal microbes from the host in their guts that contaminate plate cultures and may support growth of free-living stages in early phases of proliferation or detract from it when overgrowth occurs. These biological similarities have facilitated adaptation of DNA transformation technology, particularly gonadal microinjection, from *C. elegans* to *Strongyloides* and *Parastrongyloides*.

### Gonadal microinjection

The first step towards adaptation of standard microinjection-based techniques for DNA transfer to *Strongyloides* took place when expression of a transcriptional reporter fusing the promoter for the cellular actin-encoding gene *Ss-act-2* to the *gfp* coding sequence was detected in multiple embryos following a single microinjection of the encoding plasmid into the distal gonads of free-living *S. stercoralis* females (Lok and Massey, 2002). Although the precise organization of the *Strongyloides* germline was not known at that time, this result was consistent with the microinjection having delivered the transgene constructs into the cytoplasm of a gonadal syncytium as occurs in *C. elegans*, resulting in transformation of multiple oocyte nuclei, and establishing microinjection as a viable means of gene transfer in this parasite and transgene-encoded green fluorescent protein (GFP) as an effective *in vivo* reporter. Initial enthusiasm about this observation (Grant, 2002), however, gave way to discouragement as transformation constructs, configured as they were with parasite-specific 5′ regulatory sequences but 3′ UTRs from *C. elegans*, were expressed only in abortively developing embryos and not in developing larvae and adult worms. This and other early attempts at transgenesis in *S. stercoralis* employed the collection of modular *C. elegans* transformation vectors (Fire *et al.* 1990), which allow incorporation of any promoter and coding sequence upstream of a multipurpose terminator such as the *unc-54* 3′ UTR. It was not until a parasite specific 3′ UTR, that of *Ss-era-1*, was substituted for this *C. elegans*-derived terminator in transgene constructs that sustained, tissue-specific expression in developing larval and adult *Strongyloides* was achieved (Li *et al.* 2006).

### Transient transgene expression in S. stercoralis

The discovery that endogenous 5′ and 3′ regulatory sequences are required for sustained transgene expression throughout development opened new avenues for investigating the temporal and anatomic patterns of gene expression in *S. stercoralis*. The finding that a transcriptional reporter fusing the promoter of *Ss-era-1* to the *gfp* coding sequence is expressed in intestinal cells of transgenic first-stage larvae of *S. stercoralis* similarly to the intestinal pattern of expression of the homologous *era-1* in *C. elegans* (Li *et al.* 2006) bolstered confidence that similar transcriptional reporters could be used to infer expression patterns of other genes of interest in the parasite. In short order, reporter constructs fusing the promoters for numerous *S. stercoralis* genes to sequences encoding either GFP or a red fluorescent protein, mRFPmars, were found to be expressed in patterns similar to reporters incorporating promoters of their orthologues in *C. elegans*. Among these were constructs incorporating promoters for *Ss-gpa-3*, orthologue of an amphidial neuron-specific G protein *α* subunit in *C. elegans*, *Ss-act-2*, encoding the orthologue of a cellular actin in *C. elegans* and *Ss-rps-21*, the orthologue of a gene in *C. elegans* encoding the ubiquitously expressed ribosomal small subunit protein (Junio *et al.* 2008). It is noteworthy that although their anatomical expression patterns were unique, all of these constructs incorporated the same 3′ UTR, that of *Ss-era-1*. This indicates that like the unc-54 3′ UTR in *C. elegans*, this 3′ UTR constitutes a multifunctional terminator in *S. stercoralis* with potential utility in the design of a modular vector collection akin to that available for *C. elegans*.

### Transgenesis in P. trichosuri

*Parastrongyloides trichosuri* can undergo an apparently unlimited number of sequential free-living generations of short duration while maintaining the ability to form iL3 and establish intestinal infection in a mammalian host. Thus, *P. trichosuri* constitutes an exceedingly powerful model incorporating a parasitic nematode that is amenable to genetic study (Grant *et al.* 2006*b*). Grant and co-workers have established this model in their laboratories in Australia and New Zealand, maintaining the parasitic cycle in a natural definitive host, the brushtail possum, *Trichosurus vulpecula*. Export regulations currently restrict use of the brushtail possum to its natural geographic range in Australasia. However, discovery that *P. trichosuri* can establish a long-standing patent infection in another Australian marsupial, the sugar glider *Petaurus breviceps* (Nolan *et al.* 2007), which is bred and sold internationally in the pet trade, enables establishment of this model throughout the world. Among the many attributes of *P. trichosuri* that bolster its status as a model parasitic nematode are its capability to undergo an apparently unlimited number of sequential free-living generations with a generation time of 48–60 h at 20 °C. and its undiminished capacity to form iL3 when cultured in medium conditioned by extended cultivation of free-living larvae at high population density. These free-living generations may be grown in plates of NGM agar with 0·25 mg mL^−1^ peptone (10% of standard concentration) and supplemented with *E. coli* HB101 and an extract of host feces (Grant *et al.* 2006*b*). The possibility of extended maintenance of free-living *P. trichosuri* in a simple in vitro culture system affords the ability to perpetuate transgenic lines and to conduct crosses. Once derived, iL3 of *P. trichosuri* undertake a canonical pattern of parasitic nematode development with percutaneous infection followed by tissue migration that culminates in population of the intestinal tract with adult male and female parasites and release of eggs in host feces. Thus, the *P. trichosuri* system is invaluable in meeting the essential requirements of a formal genetic model for parasitic nematodes (Grant *et al.* 2006*b*). Transgenesis, employing the basic components of microinjection of plasmid based vectors into the syncytial ovary as developed for *C. elegans*, has been adapted for both free-living and parasitic females of *P. trichosuri* (Grant *et al.* 2006*a*). Continuous free-living culture of F1 transgenic progeny has enabled establishment of *P. trichosuri* lines that stably transmit and express transgene constructs in both the free-living and parasitic cycles. In general, although parasitic females of *P. trichosuri* may be transformed with plasmid based vectors in this manner, relatively poor survival and fecundity of these when cultured after microinjection make collection of transgenic progeny and derivation of stable lines more efficient when initial transformation is done in free-living females (Grant *et al.* 2006*a*).

Initial studies in *P. trichosuri* centred upon plasmid vectors based on the standard backbones of the modular vector library for *C. elegans* (Fire *et al.* 1990; Grant *et al.* 2006*a*). Reporter constructs incorporating promoters from either *P. trichosuri* (*Pt-hsp-1*) or *C. elegans* (p212GATA or *let-858*) fused to the *lacZ* and *gfp* coding sequences drive *β*-galactosidase expression in *P. trichosuri* in temporal and anatomical patterns that are appropriate for these promoters. However, in these initial studies, simultaneous GFP expression from these constructs was not seen, although the *gfp* coding sequence could be detected in transgenic worms through multiple rounds of culture and host passage. It was hypothesized at the time that failure by *P. trichosuri* to express GFP fluorescence is a general phenomenon in this parasite (Grant *et al.* 2006*a*), but the advantages of an *in vivo* marker for transformation, preferably one that does not impair the invasive or migratory capacity of the worm argue strongly for further exploration of transgene encoded fluorescent markers for *P. trichosuri*. One potential avenue to explore is the possibility that a parasite-specific 3′ UTR, as opposed to the *Ce-unc-54* 3′ UTR that was used in initial experiments, could enhance expression of transgene-encoded GFP in *P. trichosuri* as was the case in *S. stercoralis* (Li *et al.* 2006). Nevertheless, initial studies with transgenesis in *P. trichosuri* using the *lacZ* reporter (Grant *et al.* 2006*a*) underscore the very significant potential of this model for functional genomic study.

### Transgenesis in S. ratti

*Strongyloides ratti* is arguably the best studied of the animal parasitic Rhabditida in terms of its evolutionary (Gemmill *et al.* 2000; Dorris *et al.* 2002; Fenton *et al.* 2004), developmental (Viney, 1996; Harvey *et al.* 2000; Crook and Viney, 2005; Gardner *et al.* 2006) and reproductive biology (Viney *et al.* 1993; Viney, 1994; Harvey and Viney, 2001) and in terms of ecological aspects of its relationship with its host (Harvey *et al.* 1999; Wilkes *et al.* 2004, 2007; Paterson *et al.* 2008; O'Meara *et al.* 2010). Moreover, studies of *S. ratti* have been at the forefront of genome- and transcriptome-scale investigations of the evolution of parasitism in parasitic nematodes (Thompson *et al.* 2005, 2006, 2008, 2009; Spinner *et al.* 2012). *Strongyloides ratti* has all of the biological attributes that made adaptation of methods for transgenesis to *S. stercoralis* and the additional advantage of a natural host, the rat, which is suitable for laboratory experimentation. This and its status as a general model for parasitic nemtatode biology, made development of transgenesis in *S. ratti* a substantial imperative.

Exploration of transgenesis in *S. ratti* to date has involved microinjection of now standard vector constructs for *S. stercoralis*, including both 5′ and 3′ regulatory sequences from *S. stercoralis*, into the syncytial ovary of free-living female *S. ratti*. In an initial study (Li *et al.* 2011), two transcriptional reporter constructs were tested in *S. ratti*. These fused promoters for either the cellular actin-encoding gene *Ss-act-2* or the ribosomal small subunit-encoding gene *Ss-rps-21* to the *gfp* coding sequence with both constructs incorporating the *Ss-era-1* 3′UTR. Both constructs were expressed in a proportion of F1 larval progeny of microinjected free-living female worms. Anatomical expression patterns of the two transgenes in *S. ratti* were virtually identical to those reported for *S. stercoralis* (Junio *et al.* 2008). Expression of *Ss-act-2::gfp* was restricted to body muscle, while *Ss-rps-21::gfp* was expressed in all body tissues of *S. ratti* with a bright locus of GFP fluorescence concentrated in the genital primordia of transgenic L1, exactly as seen in *S. stercoralis*. Although anatomical expression patterns of the two constructs in *S. ratti* were identical to those seen in *S. stercoralis*, their transformation efficiency, as reflected in the proportion of F1 progeny transformed, was lower in *S. ratti* than in *S. stercoralis*. For example, *Ss-rps-21::gfp* was expressed in 33·5% of progeny of microinjected *S. stercoralis* females, but in only 5·6% of *S. ratti* progeny. This trend held for *Ss-act-2::gfp* as well. This apparent difference in penetrance of the transgenes in the two parasites could be attributable to several factors. It is plausible that although genomic homology between the *act-2* and *rps-21* promoters in the two parasites is high (identity estimates of 64·5 and 81·75%, respectively) it is insufficient to give optimal expression of GFP under the *S. stercoralis* elements in *S. ratti*. This hypothesis should be tested by comparing expression frequencies in *S. ratti* of constructs incorporating *S. stercoralis* regulatory sequences to those of constructs incorporating their homologs from *S. ratti*. It is also possible that the relative inefficiency of transformation observed in the comparison of *S. ratti* to *S. stercoralis* could be due to differences in position of the gonadal syncytium in the two parasites or in the former parasite being more ‘fragile’ to the mechanics of injection than *S. stercoralis*. It is noteworthy that members of the authors' laboratory who conduct microinjections frequently are unanimous in their impression that free-living female *S. ratti* are more ‘delicate’ and therefore more difficult to inject successfully than *S. stercoralis*. Refinements that overcome such technical barriers to efficient transformation of *S. ratti* are an important near-term research priority. These shortcomings notwithstanding, proof-of-principle for transgenesis in *S. ratti* was a significant advance given the importance of this parasite as a model organism and given its eventual practical importance in derivation of *Strongyloides* spp. lines stably transmitting and expressing chromosomally integrated transgenes, to be discussed below.

### Transient expression of plasmid-encoded transgenes

As discussed above, reporter transgenes incorporating *gfp* and encoded in plasmid vectors are expressed in promoter-regulated fashion in the tissues of larval progeny of free-living female *S. stercoralis* (Li *et al.* 2006; Junio *et al.* 2008). Although temporal as well as anatomical expression patterns of such reporter transgenes depend upon the specific promoter they incorporate, the authors' laboratories have observed that a transgene regulated by the promoter of *Ss-act-2* is expressed in pre-parasitic larval stages emanating from the transformed free-living female and in post-infective larvae and parasitic females within an experimental host the Mongolian gerbil *Meriones unguiculatus* (Nolan *et al.* 1993; Junio *et al.* 2008). This is a significant finding because it implies that transgenesis could be viable tool in studies of the biology of in pre-parasitic larvae and the biology and host interactions of post-infective larvae and parasitic females of *S. stercoralis* of the F1 generation following gene transfer.

The facts that plasmid encoded transgenes are inherited in *C. elegans* as multicopy episomal arrays and that expression of transgenes in such arrays continues undiminished through many generations of passage (Mello *et al.* 1991; Mello and Fire, 1995) prompted the authors to ascertain inheritance and expression of plasmid encoded transgenes in *S. stercoralis*. These investigations (Junio *et al.* 2008) revealed that DNA from a plasmid-encoded transgene, *Ss-act-2*p::*gfp::Ss-era-1* 3′UTR, may be detected by PCR in *S. stercoralis* for at least five passages carried out without selection and alternating between generations of parasitic females in experimentally infected gerbils and free-living adults in culture. However, the expression of this transgene, while robust in the F1 generation, cannot be detected in transgenic worms from subsequent passages. This result suggests that, in contrast to *C. elegans*, some active transgene silencing mechanism pertains in *S. stercoralis* transformed with plasmid-based vectors and subjected to rounds of host and culture passage beyond the F1 generation. The reason for this transgene silencing has not been formally studied, but the authors hypothesize that, such as *C. elegans, S. stercoralis* assembles plasmid-encoded transgenes into tandem, multi-copy, episomal arrays and that transgenes within these arrays are inherited, but silenced after the first host passage because of their highly repetitive nature, their episomal location or both. The first test of this hypothesis came with the transposon-mediated chromosomal integration of the *Ss-act-2*p::*gfp::Ss-era-1* 3′ UTR transgene in *S. ratti*.

### Stable, heritable expression of transgenes integrated into the chromosome

A substantial test of the hypothesis that silencing of plasmid-encoded transgenes in *S. stercoralis* in generations following host passage is due to their inheritance as high copy-number tandem extrachromosomal arrays was undertaken by integrating the transgene *Ss-act-2*p::*gfp::Ss-era-1* 3′UTR into the chromosome using the *piggyBac* transposon system (Shao *et al.* 2012; Lok, 2013). Testing of the *piggyBac* system, which was derived from a natural transposable element in the wax moth *Trichoplusia ni* (Cary *et al.* 1989), was prompted by its successful deployment for integrative transgenesis in *Schistosoma mansoni* (Morales *et al.* 2007). The *piggyBac* transposon is also effective for integrative transgenesis in the protozoan agents of malaria, *Plasmodium falciparum* (Balu *et al.* 2005) and *Plasmodium berghei* (Fonager *et al.* 2011).

Integration of transgenes by *piggyBac* was accomplished in *S. ratti* by co-transforming free-living female worms by gonadal microinjection with plasmid vectors encoding the *piggyBac* transposase under the ubiquitously active *Ss-rps-21* promoter (the helper plasmid) and a donor plasmid encoding the *Ss-act-2*p::*gfp::Ss-era-1* 3′UTR reporter construct by inverted terminal repeats specific for the *piggyBac* transposon (Shao *et al.* 2012). Progeny from free-living females so transformed were reared to iL3 on culture plates and used to infect susceptible rats. Feces from rats infected with first-generation transformants were screened for second-generation progeny expressing GFP beginning on day 7 of infection. The few such individuals recovered, usually <10, were reared to free-living males and females in plate culture, mass mated and their progeny screened for transgene expression by stereo fluorescence microscopy. Cohorts of transgene-expressing larvae, generally numbering <100, were reared to iL3 and inoculated into a rat. This process of alternating host and culture passage, with selection on GFP expression, resulted in the establishment of stable transgenic lines of *S. ratti*, that are in continuous rearing to date. A crucial factor in this process was the well-adapted host–parasite pairing represented by *S. ratti* in the rat, in which the efficiency of infection is ~50% allowing successful host passage of the very small numbers of transgenic iL3 available in the first three generations of line establishment (Shao *et al.* 2012). By contrast, the relatively low efficiency of *S. stercoralis* infection in the gerbil (Nolan *et al.* 1993) has prevented similar selection of stable transgenic lines of this parasite to date.

## APPLICATIONS OF TRANSGENESIS IN RESEARCH ON *STRONGYLOIDES*

### Inferring anatomical patterns of gene expression

One of the obvious applications of transgenesis in parasitic nematodes is to investigate anatomical patterns of gene expression, expecting that these will provide clues about the functions of these genes. The wealth of data on anatomical patterns of gene expression in *C. elegans* provides some basis for comparative study, assuming that similar expression patterns infer similar function. Caution is warranted in drawing such conclusions, however, given the large phylogenetic divergence of parasites in Clades II (*Trichuris* and *Trichinella*) III (Ascarid round worms and filariae) and IV (*Strongyloides* and related genera) from *C. elegans* and its parasitic relations (hookworms and other strongylids) in Clade V. Such comparisons are also limited by the large proportions of novel genes in parasitic nematodes that have no known homologs in *C. elegans*. Moreover, expression patterns of reporter transgenes provide only an indirect indication of endogenous gene expression in a given cell or tissue, as opposed to the more direct approaches such as in situ DNA–RNA hybridization.

These caveats notwithstanding, transformation of *Strongyloides* spp. and *P. trichosuri* with reporter transgenes fused to the promoters of genes of interest have provided evidence of anatomical expression patterns of these genes, sometimes revealing significant concordance with patterns of their homologs in *C. elegans*, in spite of wide phylogenetic divergence. In *P. trichosuri*, a LacZ reporter linked to the promoter of the heat shock protein-encoding gene *Pt-hsp-1* was expressed predominantly in intestinal cells of free-living adults. This pattern is consistent with anatomical expression patterns of the *C. elegans hsp-1*, the orthologue of *Pt-hsp-1* (Grant *et al.* 2006*a*).

Studies employing transgenesis in *Strongyloides stercoralis* have focused on anatomical and subcellular expression patterns and functions of genes encoding intermediates of an insulin-like signalling pathway analogous to that regulating dauer larval development in *C. elegans*. A reporter transgene incorporating a putative promoter comprising 1956 bp of 5′ flanking genomic sequence from *Ss-daf-2*, encoding an insulin-like receptor kinase orthologous to of *C. elegans daf-2*, drove weak GFP expression in the intestines of transgenic first-stage larvae of *S. stercoralis*. This pattern was virtually identical to that seen in first-stage *C. elegans* larvae transformed with a similar reporter construct incorporating the *Ce-daf-2* promoter (Massey *et al.* 2013). These results are consistent with similar patterns of DAF-2 expression in *S. stercoralis* and *C. elegans* and so with similar functions of this insulin-like receptor kinase in regulation of third-stage larval development. Enhanced GFP (EGFP) expression under a putative promoter for *Ss-age-1*, which encodes the ortholog of the insulin-regulated PI3 kinase AGE-1 in *C. elegans*, was observed in cells of the hypodermis, intestine and genital primordium, as well as in phasmidial and amphidial neurons of transgenic first-stage larvae of *S. stercoralis* (Stoltzfus *et al.* 2014). A similar EGFP reporter construct incorporating the *Ce-age-1* promoter had a comparable pattern of expression in transgenic first-stage larvae of *C. elegans*, including a striking concordance in the amphidial neurons. In *S. stercoralis, Ss-age-1* reporter expression was limited to AWC class neurons, whereas in *C. elegans*, reporter expression was observed in two neuron pairs, AWC and ASJ. In the aggregate, these data especially the similar patterns of reporter expression in amphidial neurons support similar functions for the AGE-1 PI3 kinases and for insulin-like signalling generally in *C. elegans* and *S. stercoralis*. The output of insulin-like signalling in *C. elegans* is negative regulation of the FOXO-class transcription factor DAF-16. Under conditions favouring continuous reproductive development insulin-like signalling results in phosphorylation of DAF-16 at four Akt sites, resulting in transport of the transcription factor out of the nucleus (Paradis and Ruvkun, 1998). Under dauer inducing conditions, shutdown of insulin-like signalling leaves DAF16 in an unphosphorylated state resulting in retention in the nucleus where the factor confers a dauer-specific pattern of gene expression resulting in developmental arrest and lifespan extension (Kenyon *et al.* 1993; Ogg *et al.* 1997; Tissenbaum and Ruvkun, 1998; Libina *et al.* 2003). *Caenorhabditis elegans* daf-16 was originally described as expressing two major transcripts, a and b, with isoforms *daf-16a1* and *daf-16a2* expressed under the control of the *α* promoter located 5′ of the entire coding sequence and *daf-16b* expressed under the control of the *β* promoter located within intron 4 of the gene (Ogg *et al.* 1997). Further study has revealed much additional complexity in the profiles of *daf-16* transcript isoforms. At this writing, WormBase catalogues some 12 *daf-16* isoforms that are apparently derived by alternative splicing of transcripts expressed under three promoters designated *d*/*f* (Kwon *et al.* 2010), *α* and *β* (http://www.wormbase.org/species/c_elegans/gene/WBGene00000912#0-9g-3). Significantly, the long-recognized function of *daf-16* in regulating lifespan in *C. elegans* has been attributed to a new isoform, *daf-16d/f* (Kwon *et al.* 2010). In general, the *daf-16* ortholog in *S. stercoralis, Ss-daf-16*, has similar structure to *Ce-daf-16*, albeit with fewer and smaller introns, and similar regulation of major transcripts by *d*/*f, α* and *β* promoters positioned upstream of and within the gene, respectively (Massey *et al.* 2003; Stoltzfus *et al.* 2012*b*; Hunt *et al.* 2016). However, in contrast to the numerous and diverse *Ce-daf-16* isoforms described above, only three transcripts of the parasite gene have been discovered to date. These are *Ss-daf-16a, Ss-daf-16b* and *Ss-daf-16c*, which are under control of the *α, β* and *d*/*f* promoters, respectively (Stoltzfus *et al.* 2012*b*; Hunt *et al.* 2016). Studies employing transgenesis in *S. stercoralis* have largely confirmed similar patterns of expression, subcellular trafficking and developmental function for *Ss-DAF-16b*, which was initially named FKTF-1b, and is the orthologue of *C. elegans* DAF-16b (Massey *et al.* 2003, 2006). A GFP reporter incorporating the *Ss-daf-16β* promoter was expressed broadly in transgenic larvae of *S. stercoralis*, with intense fluorescence in hypodermal cells, numerous neurons, intestine and a conspicuous cylindrical zone of the pharynx (Castelletto *et al.* 2009). This pattern, notably including zonal expression in the pharynx, was virtually identical to expression patterns of a similar reporter incorporating the *Ce-daf-16β* promoter in transgenic first-stage larvae of *C. elegans* (Lee *et al.* 2001). Highly similar anatomical patterns of *daf-16β* promoter activity in *C. elegans* and *S. stercoralis*, encompassing organs and tissues remodelled during morphogenesis of both dauer larvae and iL3, respectively, is additional evidence of conserved function of the insulin-regulated forkhead transcription factors encoded by *Ce-daf-16* and *Ss-daf-16*.

RIO kinases constitute a class of atypical protein kinases essential for basic cellular functions in organisms ranging in complexity from archaea to humans. Specific members of this class are either conserved through this entire phylogenetic range (e.g. RIOK-1) or restricted to metazoans (e.g. RIOK-3). Homologues of RIOK-1 are necessary for chromosome maintenance, cell cycle control, and cleavage and maturation of ribosomal subunit proteins (LaRonde-LeBlanc and Wlodawer, 2005*a*, *b*). *Ce-riok-1* is conserved in parasitic nematodes. Transcriptional silencing of this gene by RNAi brings about embryonic lethality and impairment of larval development in *C. elegans*, indicating that this protein kinase is necessary for nematode development (Hu *et al.* 2008). A gene encoding RIOK-1 is conserved in the genome of *S. stercoralis* (Yuan *et al.* 2014*b*). A reporter construct fusing a putative promoter comprising 4280 bp of 5′ flanking sequence from *Ss-riok-1* to the *gfp* coding sequence was expressed in body wall and pharyngeal muscle of newly hatched transgenic first-stage larvae of *S. stercoralis*. In older post free-living larvae, a strong neuronal pattern of *Ss-riok-1* reporter expression emerged with bright GFP signal seen in head and phasmidial neurons, as well as in longitudinal and body neuron tracts and their interconnecting commissures (Yuan *et al.* 2014*b*). Localization of *Ss-riok-1* reporter expression to body muscle of developing larvae and to newly forming nerve tracts in these worms is consistent with the conserved functions of RIOK-1 homologs in control of ribosome assembly, cell cycling and development in eukaryotic cells. Another atypical protein kinase in the RIO family, RIOK-3, is conserved in *S. stercoralis* (Yuan *et al.* 2014*a*). In contrast to the highly conserved RIOK-1s, RIOK-3s are only encoded in the genomes of metazoan organisms (LaRonde-LeBlanc and Wlodawer, 2005*a*). While they appear to be associated with 40s ribosomal subunits in human cells, the function of RIOK-3s in ribosomal biogenesis is obscure. More apparent is their function in the cycling and proliferation of human cancer cells (Stephens *et al.* 2005; Kimmelman *et al.* 2008; Shan *et al.* 2009). An investigation into the hitherto unknown structure, phylogenetic relationships and function of *Ss-riok-3* (Yuan *et al.* 2014*a*) employed transgenesis to study anatomical expression patterns of this gene. A reporter construct fusing an 881 bp putative *Ss-riok-3* promoter to the coding sequence of *gfp* was expressed in intestinal cells and in head neurons of transgenic post free-living first- and second-stage larvae of *S. stercoralis*. This pattern changed markedly during subsequent development such that iL3 expressed the *Ss-riok-3* reporter predominantly in body wall muscle. While intestinal expression of RIOK-3 is conserved in *S. stercoralis* and *C. elegans* (McKay *et al.* 2003; Hunt-Newbury *et al.* 2007; Yuan *et al.* 2014*a*), expression in head neurons and the transition to expression in body wall muscle in iL3 were only seen in *S. stercoralis* and may indicate functions of *Ss-RIOK-3* that are specific to parasitism, such as sensing the environment for host cues and preparation of muscles necessary for invasion of the host and migration therein (Yuan *et al.* 2014*a*). Overall, findings on anatomical expression of *Ss-riok-1* and *Ss-riok-3* support further investigation into the suitability of RIO kinases as anthelmintic targets.

### Observing patterns and confirming mechanisms of intracellular protein trafficking

To date, reporter constructs used in all but one study of anatomical gene expression patterns in *Strongyloides* and *Parastrongyloides* have constituted simple fusions of coding sequences of LacZ (Grant *et al.* 2006*a*), *gfp* (Grant *et al.* 2006*a*; Li *et al.* 2006, 2011; Junio *et al.* 2008; Massey *et al.* 2013; Yuan *et al.* 2014*a*, *b*) or egfp (Stoltzfus *et al.* 2012*a*, 2014) to putative promoters for the genes of interest. In contrast to these, a study employing transgenesis to investigate the function of *Ss-daf-16* (previously named *fktf-1*) employed a construct designed to express an N-terminal fusion of GFP with wild-type *Ss*-DAF-16b (Castelletto *et al.* 2009). Expression of this GFP-tagged protein allowed patterns of its cytoplasmic and nuclear localization within hypodermal cells to be assessed in living *S. stercoralis*. This assessment was crucial because the activities of DAF-16 orthologues in nematodes and other eukaryotic FOXO transcription factors are regulated by phosphorylation-dependent nuclear export as a function of insulin-like signalling (Cahill *et al.* 2001). Hypodermal cells of post free-living larvae expressing GFP::Ss-DAF-16b under the *Ss-daf-16β* promoter exhibited fluorescence in both nucleus and cytoplasm, but with markedly brighter intensity in the nucleus, a pattern consistent with significant nuclear localization of the transcription factor and with its function in determining the worms' consequent developmental fate as dauer-like iL3. Mutations conferring substitutions predicted to block phosphorylation of four AKT sites on *Ss*-DAF-16b (T22A/S238A/S240A/S317A) resulted in constitutive nuclear localization of the resulting GFP fusion protein. Mutations conferring phospho-mimetic substitutions at two AKT sites on *Ss-DAF-16b* (S238E/T240E) resulted in constitutive cytoplasmic localization of the resulting GFP fusion protein. Intracellular trafficking patterns of these phosphorylation site mutant forms of Ss-DAF-16b support conserved negative regulation of this crucial developmental factor by insulin-dependent phosphorylation (Castelletto *et al.* 2009).

### Directly interrogating gene function

Predictably altering the intracellular trafficking of transgene encoded GFP::Ss-DAF-16b in *S. stercoralis*, as related in the previous section, provided the basis for expressing forms of this fusion protein designed to exert dominant interfering effects on the function of endogenous Ss-DAF-16 and assessing resultant developmental phenotypes in post free-living larvae (Castelletto *et al.* 2009). Specifically, transgene constructs encoding ‘phospho-null’ phosphorylation site mutations that resulted in constitutive nuclear localization were further mutated to delete transactivating motifs in the C-terminal domain of Ss-DAF-16b. It was reasoned that the combination of constitutive nuclear localization of the overexpressed fusion protein, now deficient in its transactivating function, would result in a dominant interfering effect on endogenous Ss-DAF-16b, which is expressed at varying levels throughout the life cycle of *S. stercoralis*. Expression of this putative dominant interfering construct in post free-living *S. stercoralis* larvae resulted in several phenotypes that were consistent with a requirement for Ss-DAF-16b in formation of iL3. These phenotypes included disappearance of secretory granules that are numerous in the intestinal cells of wild-type larvae fated for developmental arrest as iL3, retention of rhabditiform pharyngeal morphology in L3 expressing the transgene and initiation of an L3–L4 moult by these transgenic larvae in plate culture at 22 °C (Castelletto *et al.* 2009). Overall, these results, which, to our knowledge, remain the only ones derived from expression of a mutant transgene in a parasitic nematode, support the ‘dauer hypothesis’, which holds that parasitic nematodes have appropriated cellular signal transduction pathways that regulate formation of dauer larvae in free-living nematodes to regulate development of iL3 prior to and during the infective process (Hotez *et al.* 1993; Crook, 2014).

## FUTURE DIRECTIONS

### Gene disruption and editing via CRISPR/Cas9

Deploying RNA-guided nucleases based on CRISPR to introduce insertions or deletions (indels) in target genomic sites represents the state of the art in genome editing and gene disruption in eukaryotic cells and whole organisms (Pennisi, 2013). The most widely used version of this approach, hereafter referred to as the CRISPR/Cas9 system, involves mutagenesis catalysed by a complex of the *Streptococcus pyogenes* CRISPR-associated (Cas) endonuclease Cas9 and a small guide RNA (sgRNA) comprising transactivating and sequence-specific targeting domains. The CRISPR/Cas9 system has been adapted for use in non-parasitic nematodes *C. elegans* (Friedland *et al.* 2013; Lo *et al.* 2013; Waaijers *et al.* 2013), the dioecious *Caenorhabditis* species 9 (Lo *et al.* 2013) and the necromenic nematode *Pristionchus pacificus* (Lo *et al.* 2013; Witte *et al.* 2015). Transgenesis in *Strongyloides* and related genera opens the possibility of using it as a functional genomic tool in these parasitic nematodes.

**Fig. 1:**
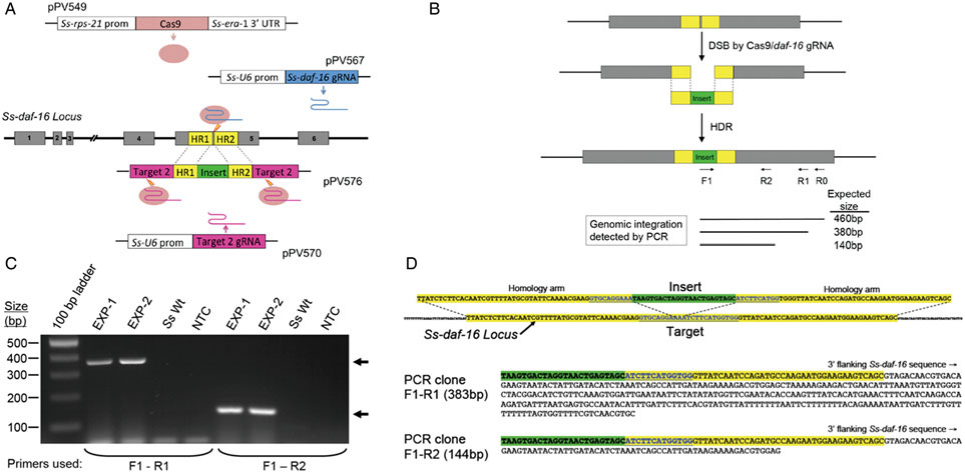
Target specific insertional mutagenesis in *S. stercoralis* via CRISPR. (A) Basic scheme involving transformation of P0 free-living female worms with plasmids pPV549 encoding Cas9, codon optimized for the extremely AT-rich genome of *S. stercoralis* (Massey *et al.* 2001; Hunt *et al.* 2016), pPV567 encoding sgRNA specific for exon 5 of *Ss-daf-16*, pPV576 encoding the 24 bp insert sequence, containing stop codons in all reading frames, flanked by 50 bp homology arms complementary to homology regions HR1 and HR2, respectively, in the target and copies of Target Sequence 2 for excision by CRISPR and pPV570 encoding Target 2-specific sgRNA. (B) Detail of target for double stranded break (DSB) in *Ss-daf-16* followed by incorporation of insert via homology directed repair (HDR). Insert contains a novel forward priming site F1. Priming sites R0-R2 in flanking genomic region for nested PCR assay are indicated, with expected sizes of products. (C) PCR products (arrows at right) from nested PCR reactions involving primer pairs F1-R1 and F1-R2. Data are from two biological replicates (EXP-1, EXP-2) with control reactions using wild type gDNA (Ss Wt) and no-template controls (NTC). Note concordance with expected product sizes. The primary reaction with F1-R0 yielded no detectable products, but did so when concentrated and used in subsequent nested reactions. (D) Sequences of *Ss-daf-16* target region (blue characters) and insert (green highlight) with homology arms (yellow highlight) and of cloned products of F1–R1 and F1–R2 reactions showing insert and with homology arm and 5′ flanking genomic sequence (unhighlighted black characters). GenBank accession numbers for constructs: pPV549 – KU665998, pPV567 – KU665999, pPV570 – KU666000, pPV576 – KU666001.

We have recently have achieved proof of principle for targeted mutagenesis in *S. stercoralis* via CRISPR/Cas9. As a target gene in this exploratory study, we selected *Ss-daf-16*, which, as discussed above, encodes an insulin-regulated FOXO transcription factor homologous to the dauer regulatory factor *C. elegans* DAF-16. We employed CRISPR/Cas9 to insert a 24 bp oligonucleotide, containing stop codons in all reading frames, into exon 5 of *Ss-daf-16*, which is expressed in all message isoforms (Fig. 1A). The insertion, therefore, should result in a null mutation. We transformed free-living female worms with plasmids encoding the Cas9 nuclease codon optimized for *S. stercoralis*, the insert with homology arms for regions flanking the expected double stranded break (DSB) in the target, and guide RNAs specific for the primary target in *Ss-daf-16* and for flanking targets in the insert donor plasmid, serving to excise the insert and homology arms designed to mediate homology directed repair (HDR) (Fig 1A). The insert contained a novel priming site that we used with three nested reverse priming sites in the 3′ flanking region of the target locus (Fig. 1B). Nested PCR from secondary and tertiary reactions yielded products of the expected size (380 and 140 bp) in two independent experiments (Fig. 1C). Sequencing these products revealed the oligonucleotide insert at the DSB flanked 5′ by *Ss-daf-16* genomic sequence with lengths expected of the products of the aforementioned nested PCR reactions (Fig. 1D). This initial study was carried out without the benefit of a visual marker for transformation, making it impossible to associate larval development phenotypes with the CRISPR-induced mutation in *Ss-daf-16*.

The preliminary result we report here is significant in demonstrating the potential for genome editing and/or gene disruption via CRISPR/Cas9 in *S. stercoralis*. However, much remains to be accomplished before this approach is realized as an effective tool for functional genomic study. Based on observations in *C. elegans* (Friedland *et al.* 2013) it is likely that the first-generation *S. stercoralis* larvae described above were heterozygous for the CRISPR/Cas9-mediated insertion in *Ss-daf-16* and that this will be the case with any first-generation *Strongyloides* spp. larvae with CRISPR/Cas9-induced mutations. A paramount goal in adapting the CRISPR/Cas9 system in *Strongyloides* spp. is to develop a practical method for rendering such mutations homozygous. This process is relatively straightforward in *C. elegans* owing to the fact of self-fertilization by hermaphrodites of this species. This factor ensures that one-fourth of the progeny of worms heterozygous for the mutation in the first-generation following transformation of parental hermaphrodites will be homozygous for the mutation. *Caenorhabditis elegans* lines homozygous for the mutation can then be derived easily by singling second-generation individuals for subsequent rearing, genotyping and phenotypic analysis (Friedland *et al.* 2013). By contrast, deriving homozygotic mutants in *Strongyloides* spp. through crossing will involve a complicated process of alternating host and culture passage, likely involving small numbers of transformed worms and encompassing a minimum of two months' time. All first-generation CRISPR/Cas9 mutants will have to be subjected to a host passage, in which they will develop to parasitic females that reproduce parthenogenetically. Isogenic progeny of these parasitic females will need to be recovered in host feces and then reared to free-living adults for crossing in agar plate culture. An *in vivo* marker for CRISPR/Cas9 mutation, such as a broadly expressed fluorescent reporter, will be crucial for this process. Second-generation mutant progeny resulting from this *in vitro* passage, which will likely number <100 but could include some homozygotic individuals, which will have to be subjected to a host passage to expand their numbers. Theoretically, stabilization of homozygotic mutant lines would then be accomplished by further alternating rounds of culture and host passage. This formally possible but difficult crossing process might be obviated by a recently described method for rendering heterozygotic CRISPR/Cas9 mutations homozygotic in a single generation through a process called the mutagenic chain reaction (Gantz and Bier, 2015). The mutagenic chain reaction involves disrupting a gene by CRISPR/Cas9-mediated insertion of an autocatalytic cassette, encoding both the Cas9 and the original targeting sgRNA under appropriate promoters, into a target locus facilitated by HDR. When expressed in heterozogotes, this autocatalytic cassette would target the wild-type allele, again creating the appropriate DSB and inserting the CRISPR/Cas9 cassette via HDR and rendering the insertional mutation homozygous within a single generation. Evaluating this system in *Strongyloides* spp. or *P. trichosuri* should be a near-term priority.

Although the CRISPR/Cas9 system provides a high level of specificity to target genes, it is imperative, especially when adapting it to a new organism, to assess the potential for off-target effects. Among other things, such effects could occur due to rare mispairing between bases in the sgRNA and the genomic target or to inadvertent similarity between the seed sequence of the sgRNA and sequences in non-target genes. Rapid advances in whole genome sequencing and the recent publication of high-quality reference genome sequences for multiple *Strongyloides* species and for *P. trichosuri* (Hunt *et al.* 2016) should allow potential off-target sites in the genomes of subject parasites to be predicted by BLAST searching with targeting domain sequences of the sgRNA. Off-target mutations to be assessed *post hoc* by aligning whole genome sequences of mutant worms with appropriate reference genomes.

As conceptualized above, CRISPR/Cas9 elements would be expressed in *Strongyloides* and related parasites either from multiple transgene constructs or from multifunctional vectors encoding two or more complete transgenes. In either case, the complexities of selecting appropriate tissue specific promoters and other regulatory elements and additional complications presented by varying temporal patterns of transgene expression and by the strong AT codon bias of *Strongyloides* and related parasite genera will likely be manifold. The need to express CRISPR/Cas9 elements from transgenes has been obviated in *C. elegans* (Cho *et al.* 2013) and more recently in the necromenic nematode *P. pacificus* (Witte *et al.* 2015) by using pre-formed Cas9/sgRNA nucleoproteins. In this approach, Cas9, which is commercially available in lyophilized form (Cho *et al.* 2013; Witte *et al.* 2015), was combined with synthetic sgRNAs and then introduced into the syncytial gonads of the worms by microinjection. In both applications, the activity of the pre-formed CRISPR/Cas9 elements was transient with target mutations appearing only in progeny produced within 9 h of microinjection in *P. pacificus*. Also, heterozygous mutations in *C. elegans* and *P. pacificus* could be rendered homozygous by self-fertilization and crossing of worms cultured through multiple generations. Finally, mutations in both *C. elegans* and *P. pacificus* could be perpetuated by serial culture passage. By contrast, crossing and multi-generation rearing of mutant lines will be more challenging in *S. stercoralis* and *S. ratti* due to the requirement for host passage in maintenance of these parasites. Nevertheless, use of pre-formed Cas9/sgRNA nucleoproteins, would eliminate many of the complexities attendant upon transgenesis in both *Strongyloides* and *Parastrongyloides* and should be explored as an alternative to the use of transgene-encoded CRISPR/Cas9 elements.

### CRISPR/Cas9-mediated transcriptional silencing and augmentation

The advent of gene disruption and editing via CRISPR/Cas9 and its demonstrated potential in parasitic nematodes also open new potentialities for experimental silencing or enhancement of transcription to infer function in genes of interest. Both of these transcriptional approaches are accomplished by using sgRNAs to guide a catalytically inactive form of Cas9 (dCas9) to a specific genomic target site (Gilbert *et al.* 2013). When bound to such target sites, dCas9 alone significantly impairs the function of RNA polymerase I, bringing about modest transcriptional silencing of the target gene. The magnitude of this effect depends to a significant degree on the site of dCas9 binding within the target gene. Binding of dCas9 near the transcriptional start site generally gives the most efficient silencing (Gilbert *et al.* 2013). Notably, the transcriptional effects of dCas9 binding to target genes may be altered many-fold by fusing transcriptional repressors or activators to the catalytically inactive enzyme. In one instance, transcriptional silencing by a dCas9 construct targeted at *gfp* in stably transformed cultured human cells was enhanced approximately 5-fold by tethering the KRAB repressor domain to the inactive enzyme. Conversely, transcription of the *gfp* encoding transgene in these same cells was increased 25-fold by fusing the transcriptional activator VP64 to dCas9. These approaches, which alternately induce transcriptional silencing (‘CRISPRi’) or activation (‘CRISPRa’) of target genes, constitute a powerful new functional genomic tool, which could be particularly important in parasitic nematodes where transcriptional silencing by RNAi is generally inefficient and often limited to a relatively small number of sensitive target genes (Dalzell *et al.* 2011; Britton *et al.* 2012).

### Conditional expression systems

Assuming that robust methods for heritable gene silencing, activation, editing and disruption can be developed for parasitic nematodes, it seems logical to project that some of the most interesting subjects for study will be genes whose functions bear upon the parasites' ability to develop to the infective larval stage and then find, invade, migrate within and ultimately maintain themselves in a mammalian host. None of the medically important parasitic nematodes can be cultured through their entire life cycles in vitro, and so host passage will be required to perpetuate transgenic lines of these organisms. It follows that it will be difficult or impossible to perpetuate lines of parasitic nematodes carrying genetic mutations or transcriptional alterations that disable these essential functions. In *C. elegans* and other free-living nematodes, null mutations in essential genes can be maintained in heterozygotic worms and then rendered homozygous by experimental crossing. While this approach may be formally possible for parasitic nematodes, it will likely be impractical in the great majority of medically important species. The need is critical, therefore, for techniques that enable conditional or regulatable transgene expression in parasitic nematodes.

**Fig. 2.**
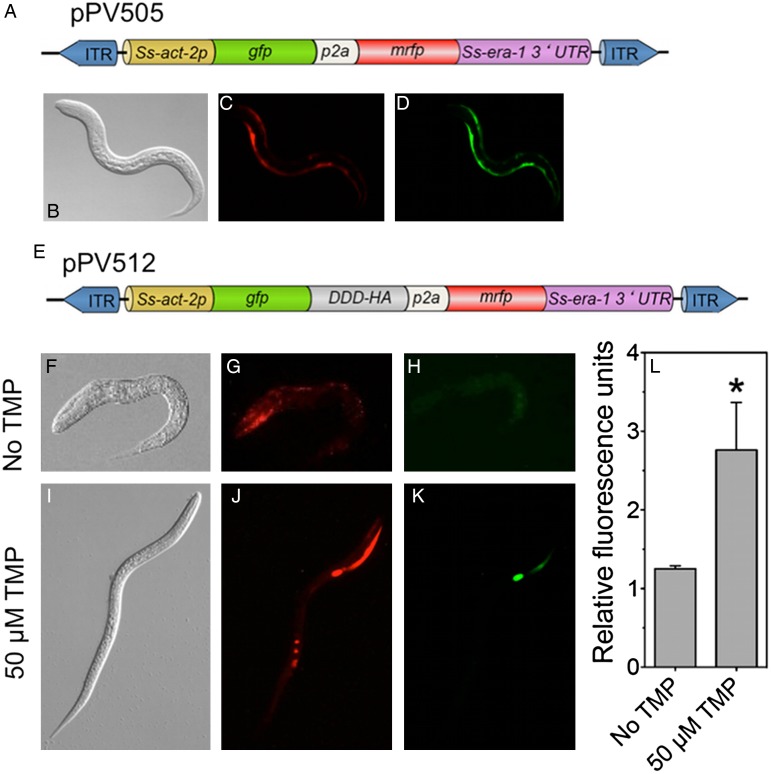
Partial regulation of *gfp* expression in *S. stercoralis* by fusion to the trimethoprim (TMP)-stabilized DHFR degradation domain. (A) Bicistronic vector pPV505 encoding *gfp* and mRFPmars with intervening self-cleaving p2a domain, all under the body wall-specific *Ss-act-2* promoter. (B–D) DIC, red and green channel images, respectively, of a post free-living first-stage larva expressing pPV505. Note uniform green and red fluorescence. (E) Bicistronic vector pPV512 identical to pPV505 except encoding *gfp*/DHFR-DD fusion. (F–H) Images of a post free-living first-stage larva expressing pPV512 and cultured without TMP. Note diminished green fluorescence. (I–K) images of an early post free-living third-stage larva expressing pPV512 and cultured with 50 *µ*m TMP. Note partial restoration of green fluorescence (K). (H) Green fluorescence quantitation in larvae expressing pPV512 and cultured with or without TMP. Means and SEM for 7 worms per sample; *Significant (*P* = 0·046) difference from ‘No TMP’ control. GenBank accession numbers for constructs: pPV505 – KU665996, pPV512 – KU665997.

One approach to this problem that has been widely adopted for mammalian systems and should be tested in nematode systems is the use of promoters with tetracycline regulatable motifs (Heinz *et al.* 2011; Ishii *et al.* 2011). In addition, regulatable transgene systems based on post translational processing and degradation of recombinant proteins in transgenic cells are also prospects. One such system that has proven effective in the malaria parasite *P. falciparum*, involves expressing recombinant proteins fused to the degradation domain of *E. coli* dihydrofolate reductase (DHFR-DD), which is stabilized in the presence of the folate analogue antibiotic trimethoprim (TMP) (Muralidharan *et al.* 2011). In the absence of TMP recombinant proteins fused to DHFR-DD are ubiquinated and thereby trafficked to the proteasome for degradation. As noted (Muralidharan *et al.* 2011), in contrast to similar systems involving degradation domains stabilized by small molecules, this system is regulated by an inexpensive and widely available antibiotic that can be safely administered to parasite-infected laboratory animals in drinking water. The authors of the present review have undertaken preliminary experiments with this TMP-regulated system in *S. stercoralis*. As a control, we prepared bicistronic construct pPV505, which encodes GFP and mRFPmars with an intervening P2A self-cleaving peptide (Kim *et al.* 2011) (Fig. 2A). This construct gave comparable levels of green and red fluorescence in transformed *S. stercoralis* larvae (Fig. 2B and D). We also prepared construct pPV512, which encodes a fusion of GFP and the DHFR-DD plus an HA tag, with mRFPmars and an intervening P2A self-cleaving peptide (Fig. 2E). In contrast to results with the control construct, *S. stercoralis* larvae transiently transformed with this construct maintained normal levels of RFP expression, but showed markedly diminished expression of GFP in the absence of TMP (Fig. 2F–H). Significantly, cultivation of *S. stercoralis* larvae transiently expressing pPV512 in 50 *µ*m TMP partially restored GFP fluorescence (Fig. 2I–K), as confirmed by Image J analysis (Fig. 2L). These data call for further studies of this TMP-regulated transgene element in *S. stercoralis* and *S. ratti* both in plate culture and in experimentally infected hosts.

Given the demonstrated potential for CRISPR/Cas9 gene editing, gene disruption and transcriptional control of genes in parasitic nematodes, it is noteworthy that a small molecule-regulated system, applicable to both CRISPR/Cas9 gene editing and to CRISPRa, has recently been developed (Zetsche *et al.* 2015). This system involves expressing Cas9 as two fragments, bearing FK506-binding protein 12 (FKBP) and the FKBP-rapamycin binding (FRB) domains, respectively. These fragments remain dissociated in the absence of rapamycin, but in the presence of the drug, they combine to reconstitute the active nuclease, which may be targeted to specific genomic sites by appropriate sgRNAs. With the split Cas9 in the presence of rapamycin, indel frequency in one target locus in human cells was 43% of that seen with wild-type Cas9, whereas indel frequency in the absence of rapamycin did not differ from non-transformed controls. In all experiments, the split Cas9 system resulted in far lower indel frequencies in validated off target sites than wild-type Cas9. In the same study, a split dCas9 system also gave significant transcriptional activation of a target locus in the presence of rapamycin when the FKBP associated fragment of the inactive nuclease was fused to the VP64 transcriptional activator (Zetsche *et al.* 2015). These approaches, involving drug inducible reconstitution of split Cas9 or dCas9 may have great potential in *Strongyloides* spp., providing that the worms can be stably transformed with constructs encoding the nuclease fragments and sgRNAs and that the worms can tolerate rapamycin at concentrations necessary to reconstitute active Cas9. In that event, free-living stages of *Strongyloides* spp. stably expressing the Cas9 fragments and appropriate sgRNAs could be treated with rapamycin to initiate gene mutation, transcriptional silencing or activation.

### New gene delivery methods

*Strongyloides* and related parasites offer a significant advantage as subjects for transgenesis in terms of the ease with which the technique of gonadal microinjection can be adapted from *C. elegans* as a means of gene delivery. However, this method cannot be adapted so readily to other medically important parasitic nematodes that lack free-living adult stages. To broaden the applicability of transgenesis and the functional genomic tools that derive from it to parasites such as hookworms, trichostrongyles, ascarids, filariae and trichurids, where access to the adult germline is currently unfeasible, it will be imperative to develop methods for gene transfer that do not rely on gonadal microinjection. As a group, such methods would obviate the need to access the adult female germline and could allow ‘hands-free’ transformation of parasitic nematodes *en masse*. One such method with theoretical potential is biolistic transfer or particle bombardment. This method has been used widely for integrative transformation in *C. elegans* (Praitis, 2006; Schweinsberg and Grant, 2013), and for transient transformation of filariae (Higazi and Unnasch, 2013) and ascarid roundworms (Davis *et al.* 1999). However, while this method has proven effective for transient expression of nucleic acid constructs, the level of mortality among bombarded parasitic nematodes has prevented its use thus far in derivation of viable, developing parasites that stably express transgenes (Xu *et al.* 2011). Also, the low frequency of transgene integration into the chromosomes of *C. elegans* following biolistic transfer can be offset by a selection process in which worms with a mutation in *unc-119* that impairs motility and dauer formation are rescued by a plasmid encoding wild-type *unc-119* co-transferred with the construct of interest (Schweinsberg and Grant, 2013). This selection strategy, although feasible in theory, is not currently available for any parasitic nematode.

Electroporation of nucleic acids has proven an effective means of transforming schistosomes (Correnti and Pearce, 2004; Correnti *et al.* 2007; Kines *et al.* 2010; Beckmann and Grevelding, 2012). The success of this approach undoubtedly stems from the structure of parasitic flatworm tegument, which constitutes a cellular membrane bounding a cytoplasmic syncytium surrounding the organism. The non-cellular cuticles of parasitic nematodes are not directly comparable to this integument, and so the parameters for transferring nucleic acids into them by electroporation are likely to differ markedly from those used for schistosomes. Nevertheless, a systematic approach, possibly drawing upon bioengineering expertise, to optimize the matrix of voltage, current, wave form and time may yet enable the use of this powerful method for gene transfer into a wide range of parasitic nematodes. It is noteworthy in this regard that double-stranded RNAs and short interfering RNAs have been electroporated successfully into pre-infective larvae of the parasitic nematode *Trichostrongylus colubriformis* (Issa *et al.* 2005).

Studies in schistosomes identify the use of pseudotyped retroviral vectors as another approach to DNA transformation (Yang *et al.* 2010; Rinaldi *et al.* 2012; Hagen *et al.* 2015). It is likely that the success of approach stems to a significant degree from the susceptibility of the schistosome tegument to invasion by the pseudotyped retroviruses and that the same caveats as discussed for electroporation will apply in the application of this approach to parasitic nematodes. Nevertheless, the nematode gut is lined by an epithelial layer that may allow entry of pseudotyped retroviruses, and so this method deserves serious scrutiny as a potential hands-free mode of DNA transformation in parasitic nematode species with free-living larval stages. Moreover, the demonstrated success of RNAi in vector stages of larval filariae (Song *et al.* 2010) argues for exploration of those stages as points of attack for transgenesis by this and other hands-free approaches to DNA transfer.

Chemically mediated DNA transformation, whereby co-precipitates of transgene DNA with calcium phosphate or transgene DNA in the presence of chemical transfectants such as polyetheneimine are taken up by competent parasite stages, has been demonstrated in schistosomes (Liang *et al.* 2013) and notably in the filaria *Brugia malayi* where heritable transformation with a luciferase reporter construct was achieved by this method (Xu *et al.* 2011). The results of this study suggest that it is crucial to expose the parasites to DNA co-precipitates during a larval moulting cycle. It is possible that the nascent cuticle exposed to the environment during this event is competent to take up the co-precipitates arguing for exploration of this method in various parasitic nematode species where larvae can be induced to undergo one or more complete moults *in vitro* or in accessible locations in mammalian hosts or vectors. Generally, this finding (Xu *et al.* 2011) argues for a major effort to adapt chemically mediated DNA transfer as a broadly applicable method for transgenesis in parasitic nematodes.

## CONCLUSION

Transgenesis is now feasible for studies of gene function in *Strongyloides* and *Parastrongyloides* using both transiently transformed parasites and worms from stable transgenic lines. These relatively new methods draw heavily on proven techniques for *C. elegans* that hinge upon microinjection of DNA constructs into the adult germline. The free-living females of *Strongyloides* and related genera represent the most advantageous points of attack for DNA transformation by this approach. Development of these methods over the past decade should soon enable powerful new techniques for gene disruption and editing and for experimental manipulation of gene expression at the transcriptional level. New data, establishing proof of principle for one such method, gene editing by CRISPR/Cas9, are provided in the context of this review. Consolidating this and related technology in *Strongyloides* and related genera will require methods for deriving homozygous mutations and maintaining them in stable lines of transgenic parasites. Accomplishing the latter aim in the cases of genes essential for development or survival will require a system for conditional or regulatable mutagenesis. Finally, while *Strongyloides* constitutes a valuable model for study of many aspects of parasitic nematode biology, it is now imperative that systems for transgenesis be developed for other parasitic nematode taxa. The chief prerequisite for a system that is broadly applicable parasitic nematodes is non-reliance on gonadal microinjection as a means of gene transfer. ‘Hands-free’ techniques such as electroporation, particle bombardment, pseudotyped retroviral vectors and chemically mediated DNA transfection should all be given due attention in the near term. Of these, chemically mediated transfection has shown the most promise to date and should receive top priority.
